# Clinical characteristics and misdiagnosis of spontaneous isolated superior mesenteric artery dissection

**DOI:** 10.1186/s12872-022-02676-9

**Published:** 2022-05-25

**Authors:** Yuanli Lei, Jinying Liu, Yi Lin, Huiping Li, Wenxing Song, Zhangping Li, Weijia Huang, Shouquan Chen

**Affiliations:** 1 Department of Emergency Medicine, The First Affiliated Hospital of Wenzhou Medical University, Wenzhou, 325000 Zhejiang People’s Republic of China; 2 Department of Radiology, The First Affiliated Hospital of Wenzhou Medical University, Wenzhou, 325000 Zhejiang People’s Republic of China; 3 Department of Gastroenterology, The First Affiliated Hospital of Wenzhou Medical University, Wenzhou, 325000 Zhejiang People’s Republic of China

**Keywords:** Spontaneous isolated superior mesenteric artery dissection, Clinical characteristics, Abdominal pain, Misdiagnosis

## Abstract

**Background:**

Spontaneous isolated superior mesenteric artery (SMA) dissection (SISMAD) is a rare disease with a potentially fatal pathology. Due to the lack of specificity of clinical characteristics and laboratory tests, misdiagnosis and missed diagnosis are often reported. Therefore, the aim of this study was to investigate the clinical characteristics and misdiagnosis of SISMAD.

**Methods:**

In a registry study from January 2013 to December 2020, 110 patients with SISMAD admitted to the First Affiliated Hospital of Wenzhou Medical University were enrolled. Descriptive methods were used to analyse clinical characteristics, laboratory data, diagnostic method or proof, misdiagnosed cases, plain computed tomography (CT) findings and dissection features. To study the relationship between dissection features and treatment modality, the selected patients were classified into the conservative group (n = 71) and the non-conservative group (n = 39). The Chi-square test and Student’s t-test were used to compare the conservative and non-conservative groups.

**Results:**

One hundred ten patients with SISMAD, including 100 (90.9%) males and 10 (9.1%) females, with a mean age of 52.4 ± 7.6 years, were enrolled in the study. Relevant associated comorbidities included a history of hypertension in 43 cases (39.1%), smoking in 46 cases (41.8%), and alcohol consumption in 34 cases (30.9%). One hundred four patients (94.5%) presented with abdominal pain. Abnormalities in the C-reactive protein lever, white blood cells count and D-dimer lever were the 3 most common abnormal findings. There were 32 misdiagnosis or missed diagnosis. Fourteen cases were misdiagnosed because of insufficient awareness. Twelve cases were misdiagnosed because of disease features. Twenty cases were misdiagnosed as SMA embolism. Among them, There were 15 cases of Yun type IIb SISMAD. Sixty-six patients underwent plain CT. The maximum SMA diameter was 12.1 (11.3–13.1) mm, and the maximum SMA diameter was located on the left renal vein (LRV) plane in 68.2% of cases. Dissection features observed on contrast-enhanced CT (CECT), CT angiography (CTA), or digital subtraction angiography (DSA) showed that there were 70 cases (63.6%) of Yun type IIb SISMAD, the maximum SMA diameter was 13.0 ± 2.4 mm, the location of the maximum SMA diameter was on the LRV plane in 64.5% of cases, and 7.3% of cases were complicated with intestinal obstruction, including bowel necrosis in 3.6% of cases. There were differences between the conservative group and non-conservative groups in the residual true lumen diameter or degree of true lumen stenosis and the presence of intestinal obstruction or bowel necrosis (all *P* < 0.05).

**Conclusion:**

For SISMAD, misdiagnosis and missed diagnosis were usually caused by insufficient awareness and disease features. SISMAD should be considered in the differential diagnosis of patients presenting with unexplained abdominal pain, especially males, patients in the 5th decade of life, patients with hypertension, and patients with an enlarged SMA diameter or a maximum SMA diameter located on the LRV plane on plain CT. Mesenteric CTA or CECT should be recommended for the investigation of these conditions.

## Introduction

**Fig. 1:**
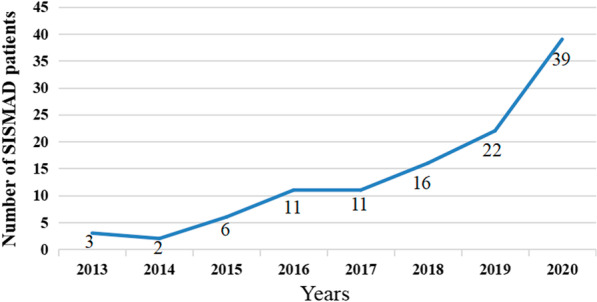
Number of SISMAD patients from 2013 to 2020. SISMAD, spontaneous isolated superior mesenteric artery dissection

Spontaneous isolated superior mesenteric artery (SMA) dissection (SISMAD), in which the involvement of the aorta is ruled out, is considered to be an uncommon vascular disease with a potentially fatal pathology [1, 2]. Bauersfeld first described this disease in 1947 [3]. From 1975 to 1999, the number of SISMAD cases rose to 23, and it reached 94 in 2009 [4]. Prior to 2014, only 622 cases were reported in China [5]. However, following improvements in diagnostic radiological practices and the wider availability of high-quality computed tomography (CT) angiography (CTA) and contrast-enhanced computed tomography (CECT) in recent years, there has been a dramatic increase in reports of this disease [1, 6, 7]. In the present study, we retrospectively collected all SISMAD cases treated at our hospital between January 2013 and December 2020, and we found that the number of cases also increased over time at our hospital (Fig. 1).

SISMAD is a rare vascular disease whose most common symptom is acute abdominal pain. The aetiology of the disease is not well known, and its clinical characteristics and laboratory data lack specificity [4, 5, 8, 9]. Plain CT is the first examination choice in the emergency department for acute abdominal pain. For SISMAD patients, the findings of plain CT often are negative or are positive but cannot explain the symptoms [6]. In some SISMAD patients, the findings show an enlarged SMA diameter and/or perivascular exudation, which are often neglected by doctors and radiologists because of insufficient awareness. Hence, SISMAD is easily misdiagnosed and missed by clinicians. Ullah et al. [4] mentioned that some SISMAD patients who presented with acute abdominal pain and underwent imaging tests were misdiagnosed with gastroenteritis, gastric pain, or nonspecific pain. Zhao et al. [10] studied 11 SISMAD patients who were admitted to the emergency department and found misdiagnosis or missed diagnosis in 7 (63.6%) of these cases.

CECT and CTA are the main clinical methods for diagnosing SISMAD in patients presenting with one of the most common symptoms (acute abdominal pain) in the emergency department [2, 4, 5, 7, 11]. CECT, as the preferred diagnostic radiological imaging modality, is recommended for patients with persistent or aggravated abdominal pain in whom the plain CT findings cannot explain the symptoms [2, 3, 9]. CECT is less expensive and very useful for the initial identification of the lesions because it minimizes partial volume artefacts and reduces the misdiagnosis [2]. CTA can quickly provide a more accurate diagnosis, especially in most cases of acute abdominal pain; additionally, CTA can clearly show the arterial dissection, dissection length, and aortomesenteric angle. CTA can even show the point of entry, the true and false lumen, and the point of re-entry, as well as the presence of thrombosis or stenosis, if present [12, 13]. It is not known which abdominal pain patients need to undergo CECT or CTA to diagnose or rule out SISMAD or which imaging modality is preferred for diagnosing unexplained abdominal pain. Luan et al. [5] investigated 589 Chinese SISMAD patients and found that 95.2% were diagnosed by CECT. Ullah et al. [4] performed a meta-analysis involving 145 cases and showed that 35.8% were diagnosed by CECT. The authors emphasized that the higher incidence of SISMAD was likely due to the introduction of CECT for investigating abdominal pain [4]. Furthermore, even though there was a lack of specificity, an enlarged SMA diameter on plain CT was the most crucial indication for diagnosis or exclusion, which is often neglected by emergency doctors and radiologists because of insufficient awareness [6]. Only a small number of studies involving few patients have included measurements of the SMA diameter [14, 15]. Additionally, the location of the maximum SMA diameter was not mentioned in the above studies. The study of the maximum SMA diameter and its location may play an important role in both choosing the diagnostic imaging modality and diagnosing SISMAD.

Thus, although reports of SISMAD have increased recently, the clinical characteristics, maximum SMA diameter and its location, diagnostic procedure, and misdiagnosis of SISMAD remain to be fully investigated in a large cohort of patients with SISMAD. To our knowledge, this is the largest single-centre series in which the clinical characteristics and misdiagnosis of SISMAD have been analysed. In the present study, we attempted to identify the clinical characteristics and imaging findings (in particular, the maximum SMA diameter and its location on plain CT) relevant to the diagnosis and misdiagnosis of SISMAD. Since abdominal pain is a very common complaint for which patients are seen in emergency settings, it is vital to highlight our results to create awareness of the possibility of SISMAD as an underlying aetiology. In short, doctors should recognize and pay attention to this rare cause of unexplained abdominal pain.

## Methods

### Study population

**Fig. 2:**
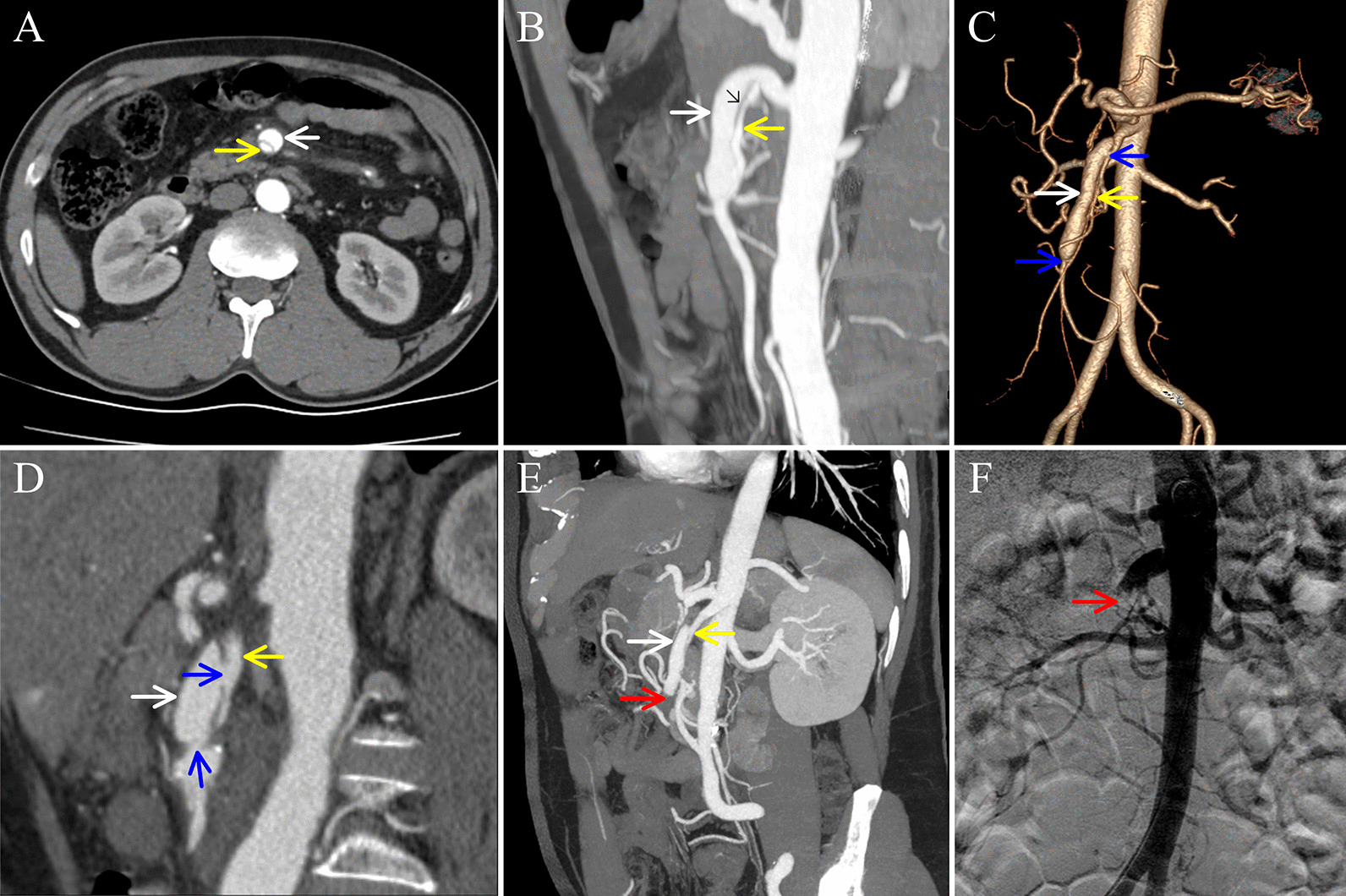
Features of Yun types I and III Dissection. (**A**) Axial CTA showing the characteristic finding of the double-lumen sign of the SMA below the LRV plane. (**B**) Sagittal CTA showing that the true and false lumens were separated by an intimal flap (black arrow). (**C**) CTA volume rendering showing the true and false lumens. (**D**) Longitudinal CTA showing the entry and re-entry sites. (**E**) Sagittal CTA showing complete occlusion of the SMA. (**F**) DSA showing complete occlusion of the SMA. The yellow arrow represents the true lumen; the white arrow represents the false lumen; the blue arrow represents the entry site or re-entry site; and the red arrow shows complete occlusion of the SMA. *CTA* computed tomography angiography, *SMA* superior mesenteric artery, *LRV* left renal vein, *DSA* digital subtraction angiography

**Fig. 3:**
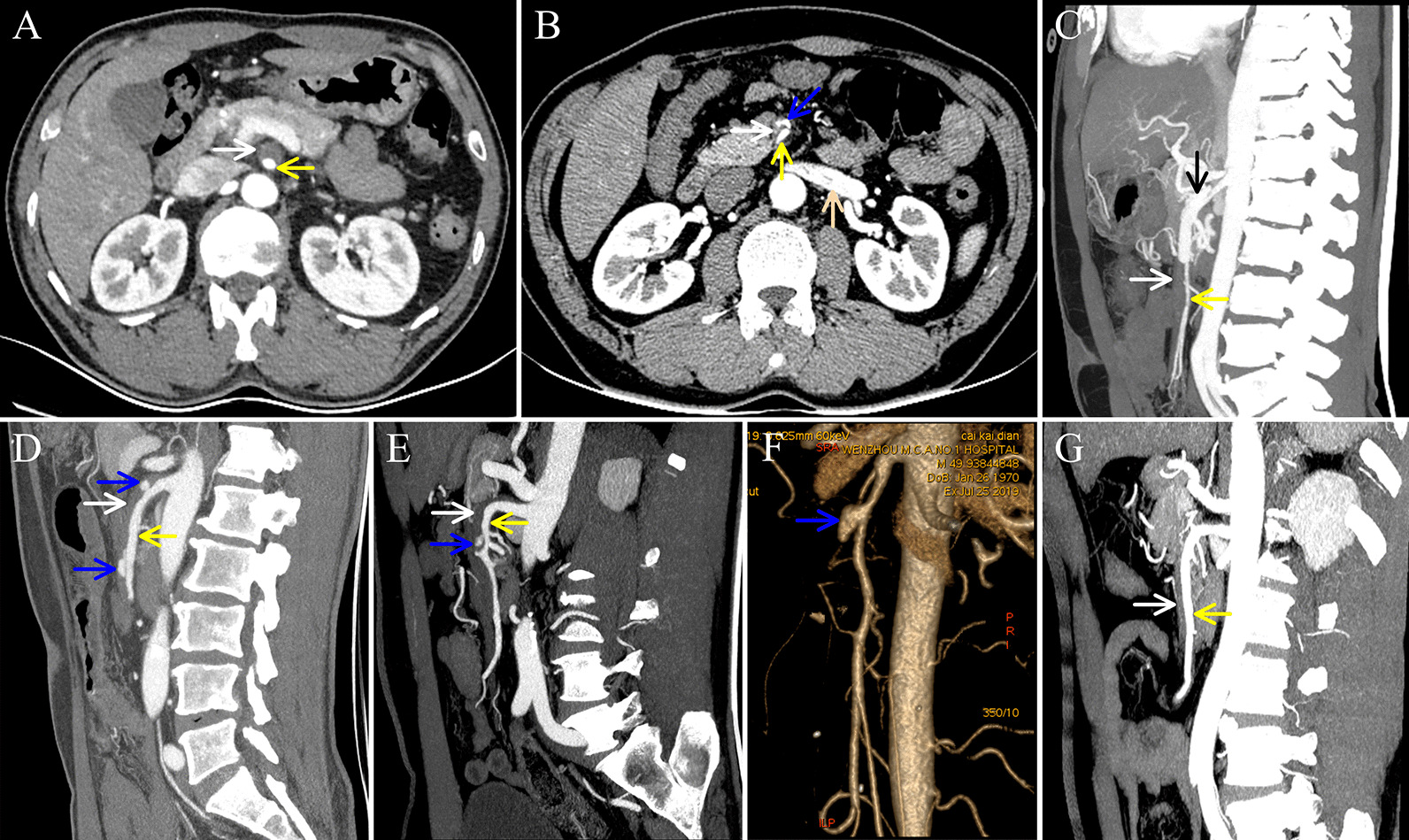
Features of Yun type IIb dissection. Axial CECT showing the true and thrombosed false lumens above the LRV plane (**A**) and the cyst-like residual false lumen in the thrombosed false lumen on the LRV (pink arrow) plane (**B**). Longitudinal CTA showing unthrombosed false lumen (black arrow) and thrombosed false lumen (**C**) and two cyst-like residual false lumens (**D**), one cyst-like residual false lumen (**E**), and no residual false lumen (**G**) in the thrombosed false lumen. CTA volume rendering (**F**) showing a cyst-like residual false lumen in the thrombosed false lumen. The yellow arrow represents the true lumen; the white arrow represents the thrombosed false lumen; and the blue arrow represents the cyst-like residual false lumen. *CECT* contrast-enhanced computed tomography, *LRV* left renal vein, *CTA* computed tomography angiography

The present study was a retrospective review of all patients who were hospitalized with a diagnosis of SISMAD according to the findings of CECT and CTA performed between January 2013 and December 2020. Two reviewers (WS and JL) independently searched the electronic medical record system to identify patients with SISMAD. Patient demographics, the duration from symptom onset to admission, clinical manifestations, comorbidities (associated risk factors), treatment modalities, and outcomes were extracted by using a prepared review data table. The study was approved by the ethics committee of The First Affiliated Hospital of Wenzhou Medical University (approval no. 2021-R013). The diagnosis of SISMAD was confirmed by one of the following signs: i) intimal flap and false lumen (Fig. 2A–E); and ii) crescent-shaped area along the wall of the SMA without contrast enhancement, indicating a thrombosed false lumen (Fig. 3). [2, 6, 16] The inclusion criteria were isolated lesions and CECT, CTA, or digital subtraction angiography (DSA) imaging data. The exclusion criteria were as follows: i) absence of CECT and CTA data; ii) asymptomatic; iii) recent abdominal trauma; and iv) concomitant aortic dissection.

### Dissection-related data collection and analysis

In our study, dissection-related information was collected through plain CT, CECT, CTA and DSA. Plain CT, CECT, and CTA were performed with a section thickness of 2.5–5 mm, 0.63–1.25 mm, and 0.3–1.25 mm, respectively. Image postprocessing methods used on the workstation included three-dimensional volume rendering, multiplanar reconstruction, curved planar reconstruction and maximum-intensity projection. The maximum SMA diameter and its location were measured on axial plain CT, CECT, and CTA views. The pathognomonic findings of SISMAD are an intimal flap and the “double-lumen sign”, which were identified on CECT CTA and DSA. Longitudinal sections revealed the entry site, dissection length, true lumen stenosis, and aortomesenteric angle. Imaging characteristics, including the distance from the ostium to the dissection entry point, dissection length, residual true lumen diameter, degree of true lumen stenosis, aortomesenteric angle, and morphologic classification of the dissection, were analysed and measured. All images were jointly reviewed by a doctor (YL) and a radiologist (YL). The measurements were repeated twice and averaged. Any discrepancies between the evaluation results were solved by discussion and a vote.

### Definitions

The degree of true lumen stenosis was calculated as (adjacent normal SMA diameter − true lumen diameter)/adjacent normal SMA diameter × 100% [11] and was divided into 3 categories: mild stenosis (< 50%); moderate stenosis (50–70%); and severe stenosis (> 70%) [1, 11, 17]. The SMA diameter was measured in 3 plane: i) above the left renal vein (LRV) plane (Fig. 3A); ii) on the LRV plane (Fig. 3B); and iii) below the LRV plane (Fig. 2A). LRV cannot be observed either above or below the LRV plane. The maximum SAM diameter was defined as the largest diameter of the aforementioned 3 plane. The location of the maximum SMA diameter was defined as the location where the maximum SMA diameter was measured. The aortomesenteric angle was defined as the angle between the axis of the aorta and the median line drawn along the SMA near the origin [18]. We classified SISMAD according to the Yun classification [19], which is based on radiological findings, in particular, the presence of true lumen patency and false luminal flow at the dissected segment. The SISMAD was categorized as follows: type I, patent true and false lumens showing entry and re-entry sites (Fig. 2B–D); type II, patent true lumen but no re-entry flow from the false lumen; IIa, visible false lumen but no visible re-entry site (blind pouch of false lumen); IIb, no visible false luminal flow (thrombosed false lumen), which usually causes true luminal narrowing (Fig. 3); and type III: SMA dissection with SMA occlusion (Fig. 2E, F). SMA occlusion was defined as occlusion of the main trunk of the SMA between the origin of the SMA and the origin of the ileocolic branch [14].

### Statistical analysis

Data were analysed using the statistical program SPSS version 18.0 (SPSS, Inc.). Numerical data are expressed as the mean ± standard deviation or median (interquartile range), and categorical data are expressed as n (%). Student’s t-test was used for numerical variables, and Chi-square test was used for categorical variables. The Chi-square test (all theoretical frequency ≥ 5), continuity-adjusted Chi-square test (1 ≤ minimum theoretical frequency < 5), Fisher’s exact test (minimum theoretical frequency < 1), and Student’s t-test were used to compare the conservative and non-conservative groups. A two-sided P < 0.05 was considered to indicate statistical significance.

## Results

### Basic information and laboratory data

A total of 110 patients with SISMAD, including 100 (90.9%) males and 10 (9.1%) females, were enrolled in the present study. The mean age of the patients was 52.4 ± 7.6 years (range 37–80 years). Among all patients, 44 (40.0%) underwent CECT, 99 (90.0%) underwent CTA (including 10 (9.1%) who underwent abdominal aortic CTA), and 55 (50.0%) underwent DSA at our hospital. The general demographic and clinical characteristics are summarized in Table 1. Relevant associated comorbidities included a history of hypertension in 43 cases (39.1%), smoking in 46 cases (41.8%), and alcohol consumption in 34 cases (30.9%). In all, 104 patients (94.5%) presented with abdominal pain. The mean time from the onset of symptoms to hospital admission was 1 (0.4–3.0) day. Apart from SMA dissections, other acute abdominal conditions included urinary calculi in 14 cases (12.7%) and intestinal obstruction in 8 cases (7.3%). The laboratory data are listed in Table 2. Abnormalities in the C-reactive protein level, white blood cell count and D-dimer level were the 3 most common abnormal findings.

**Table 1:** Clinical characteristics of SISMAD (N = 110)

Variables	N (%)
Comorbidities	
Hypertension	43 (39.1)
Liver-relative disease	15 (13.6)
Chronic hepatitis B	7 (6.4)
Hepatic adipose infiltration	6 (5.5)
Alcoholic liver disease	1 (0.9)
Hepatic insufficiency	1 (0.9)
Lung mass	4 (3.6)
Gout	4 (3.6)
Sinus bradycardia	4 (3.6)
Right bundle branch block	2 (1.8)
Hyperlipemia	2 (1.8)
Diabetes mellitus	0 (0.0)
Smoking	46 (41.8)
Alcohol consumption	34 (30.9)
Clinical symptoms	
Duration (d)	
0.04–60	1 (0.4–3.0)
Abdominal pain	104 (94.5)
Back pain	6 (5.5)
Low back pain	6 (5.5)
Chest pain	2 (1.8)
Systolic pressure ≥ 140 mmHg during the first hospital visit	59 (53.6)
Diastolic pressure ≥ 90 mmHg during the first hospital visit	55 (50.0)
Acute abdomen	35 (31.8)
Urinary calculi	14 (12.7)
Gallstone/cholecystitis	9 (8.2)
Intestinal obstruction	8 (7.3)
Bowel necrosis	4 (3.6)
Ischemic enteropathy	3 (2.7)
Appendicitis	2 (1.8)
Acute gastroenteritis	2 (1.8)
Others*	3 (2.7)

*SISMAD* spontaneous isolated superior mesenteric artery dissection

*Others included pancreatitis, inguinal hernia, and enteric infection

**Table 2:** Laboratory data in cases of SISMAD

Variables	Total		Abnormal findings*		Normal range
	N	Values	N (%)	Values	Normal range
C-reactive protein (mg/l)	81	13.2 (5–29.3)	47 (58.0)	25.2 (16.2–39.6)	0.00–10.00
White blood cells (× 10^9^/l)	110	9.93 ± 3.71	50 (45.5)	13.05 ± 3.10	3.50–9.50
D-dimer (mg/l)	102	0.38 (0.24–0.74)	34 (33.3)	1.04 ± 0.38	0.00–0.50
Total cholesterol (mmol/l)	105	4.81 ± 1.15	34 (32.4)	6.16 ± 0.85	2.44–5.17
Glycerin trilaurate (mmol/l)	108	1.25 (0.98–1.90)	33 (30.6)	2.73 ± 1.38	0.40–1.70
Low density lipoprotein cholesterol (mmol/l)	108	2.75 ± 0.90	33 (30.6)	3.82 ± 0.65	2.07–3.10
			24 (22.2)	1.72 ± 0.36	
Serum creatinine (μmol/l)	110	70.12 ± 14.91	23 (20.9)	50.87 ± 5.83	58–110
Serum lactate (mmol/l)	43	1.71 ± 0.97	9 (20.9)	3.30 ± 0.82	0.7–2.1
Serum Amylase (u/l)	69	80.20 ± 27.55	13 (18.8)	121.85 ± 21.74	28–100
Troponin I (μg/l)	63	0.002 (0.001–0.006)	1 (1.6)	1.17	0.000–0.150

Values are expressed as median (interquartile range) or mean ± standard deviation

*SISMAD* spontaneous isolated superior mesenteric artery dissection

^*^Abnormal findings: all of the variables were either above the normal upper limit or below the normal lower limit, except low-density lipoprotein cholesterol (33 values were above the normal upper limit and 24 values were below the normal lower limit)

### Diagnosis and misdiagnosis of SISMAD

The diagnostic method for or proof of SISMAD in 110 cases shown in Table 3. Forty-one cases were diagnosed because SMA disease was identified previously at another hospital; 10 cases were diagnosed because aortic dissection was suspected; 17 cases were diagnosed because plain CT showed changes in the SMA; and 22 cases were diagnosed because plain CT was negative. Among the 110 cases of SISMAD, there were 32 cases of misdiagnosis or missed diagnosis (Table 4). Fourteen cases were misdiagnosed because of insufficient awareness. Twelve cases were misdiagnosed because of disease features. Take Yun type IIb SISMAD as an example for distinguishing between insufficient awareness and disease features. There were four subtypes of type IIb in our study (Fig. 3C–G). If the longitudinal sections in these cases were similar to those in Fig. 3D–F, we classified them as cases of misdiagnosis because of insufficient awareness; if the longitudinal sections in these cases were similar to that in Fig. 3G, we classified them as cases of misdiagnosis because of disease features. No cases were misdiagnosed because the longitudinal sections were similar to that in Fig. 3C. In total, 20 cases of SISMAD were misdiagnosed as SMA embolism. Among them, there were 15 cases of Yun type IIb SISMAD, including 8 cases with longitudinal sections on CECT or CTA similar to that in Fig. 3G.

**Table 3:** Diagnostic method for or proof of SISMAD (N = 110)

Diagnostic method for or proof of SISMAD	N (%)
Imaging examination at another hospital showed disease of the SMA	41 (37.3)
Suspected aortic dissection; patient underwent abdominal aortic CTA	10 (9.1)
Plain CT showed changes to the SMA; patient underwent CTA	17 (15.5)
Plain CT findings suggested that CECT should be performed next	3 (2.7)
Plain CT findings could not explain the clinical symptoms; patient underwent CECT	35 (31.8)
Plain CT findings were negative	22 (20.0)
Plain CT findings were positive but did not include the above findings	13 (11.8)
Other*	4 (3.6)

^*^Other: One patient was diagnosed with scapulohumeral periarthritis and then underwent positron emission tomography-CT, the findings of which showed SISMAD. Three patients complained of recurrent abdominal pain and were diagnosed with gastrointestinal dysfunction. These patients underwent gastroenterological endoscopy; however, the findings were negative, so they underwent CECT or CTA. *SISMAD* spontaneous isolated superior mesenteric artery dissection, *SMA* superior mesenteric artery, *CTA* computed tomography angiography, *CT* computed tomography, *CECT* contrast-enhanced computed tomography, *DSA* digital subtraction angiography

**Table 4:** Analysis of misdiagnosis and missed diagnosis (N = 32)

Type	Cause classification	Diseases of misdiagnosis	Imaging tests with reporting problems	Yun Classification	N (%)
Misdiagnosis (N = 28)	Insufficient awareness (N = 14)	Gastrointestinal dysfunction	–	IIb	3 (9.4)
		Urinary calculi	–	IIb	1 (3.1)
				III	1 (3.1)
		Acute gastritis	–	IIb	1 (3.1)
		Scapulohumeral periarthritis	–	IIb	1 (3.1)
		Mesenteric vein thrombosis	CECT	IIa	1 (3.1)
		SMA embolism	CECT	IIb	4 (12.5)
			CECT	III	1 (3.1)
			CTA	IIb	1 (3.1)
	Disease features (N = 12)	SMA embolism	CECT	IIa	1 (3.1)
				IIb	6 (18.8)
				III	2 (6.3)
			CTA	IIb	1 (3.1)
				III	1 (3.1)
			Abdominal aortic CTA	IIb	1 (3.1)
	Imaging quality (N = 2)	SMA embolism	CECT	IIb	1 (3.1)
			CTA		1 (3.1)
Missed diagnosis (N = 4)	Insufficient awareness (N = 3)		CECT	I	1 (3.1)
			CECT	IIb	1 (3.1)
			CECT	III	1 (3.1)
	Disease features (N = 1)		CTA	IIa	1 (3.1)

*SMA* superior mesenteric artery, *CECT* contrast-enhanced computed tomography, *CTA* computed tomography angiography

### Dissection features and treatment modalities

Sixty-six SISMAD patients underwent plain CT at our hospital. Dissection features on plain CT are shown in Table 5. Dissection features observed on CECT, CTA, or DSA showed that the mean length of SMA dissection was 91.9 ± 33.1 mm, the mean distance from the ostium to the dissection entry point was 15.1 ± 9.1 mm, there were 70 cases (63.6%) of Yun type IIb SISMAD, the maximum SMA diameter was 13.0 ± 2.4 mm, the location of the maximum SMA diameter was on the LRV plane in 64.5% of cases, and 7.3% of cases were complicated with intestinal obstruction, including bowel necrosis in 3.6% of cases (Table 6). To study the relationship between the dissection features and treatment modality, the 110 cases of SISMAD were classified into the conservative group (71 cases) and the non-conservative group (39 cases) (Table 7). There were differences between the conservative and non-conservative groups in the residual true lumen diameter or degree of true lumen stenosis and the presence of intestinal obstruction or bowel necrosis (all P < 0.05; Table 6).

**Table 5:** Dissection features on plain CT (N = 66)

	Maximum SMA diameter (mm)	Location of maximum SMA diameter			Perivascular exudation
		Above the LRV plane	on the LRV plane	Below the LRV plane	
Values	12.1 (11.3–13.1)	18 (27.3)	45 (68.2)	3 (4.5)	44 (66.7)

Values are expressed as median (interquartile range) or the no. (%)

*CT* computed tomography, *SMA* superior mesenteric artery, *LRV* left renal vein

**Table 6:** Relationship between dissection features and treatment modality (N = 110)

Variables	N (%)	Conservative group (n = 71)	Non-conservative group (n = 39)	t/χ^2^	P-value
Ostium to dissection entry, mm	15.1 ± 9.1	15.4 ± 8.9	14.4 ± 9.7	0.555	0.580
Dissection length, mm	91.9 ± 33.1	93.0 ± 30.3	89.7 ± 37.9	0.501	0.618
Residual true lumen diameter, mm	2.7 ± 1.6	2.9 ± 1.6	2.3 ± 1.6	2.069	0.041
Degree of true lumen stenosis, %				8.929	0.012
< 50%	43 (39.1)	35 (49.3)	8 (20.5)		
≥ 50% and ≤ 70%	32 (29.1)	18 (25.4)	14 (35.9)		
> 70%	35 (31.8)	18 (25.4)	17 (43.6)		
Maximum SMA diameter, mm	13.0 ± 2.4	13.2 ± 2.5	12.6 ± 2.2	1.401	0.164
Location of maximum SMA diameter				0.959	0.619
Above the LRV plane	35 (31.8)	21 (29.6)	14 (35.9)		
on the LRV plane	71 (64.5)	48 (67.6)	23 (59.0)		
Below the LRV plane	4 (3.6)	2 (2.8)	2 (5.1)		
Aortomesenteric angle, °	76.3 ± 25.0	75.6 ± 25.0	77.6 ± 25.1	− 0.404	0.687
Classification				1.732	0.630
I	16 (14.5)	10 (14.1)	6 (15.4)		
IIa	6 (5.5)	5 (7.0)	1 (2.6)		
IIb	70 (63.6)	46 (64.8)	24 (61.5)		
III	18 (16.4)	10 (14.1)	8 (20.5)		
Celiac trunk dissection	8 (7.3)	5 (7.0)	3 (7.7)	0.000	1.000
Iliac artery dissection	2 (1.8)	1 (1.4)	1 (2.6)	–	1.000
Renal artery dissection	1 (0.9)	1 (1.4)	0 (0.0)	–	1.000
Intestinal obstruction	8 (7.3)	2 (2.8)	6 (15.4)	4.179	0.041
Bowel necrosis	4 (3.6)	0 (0.0)	4 (10.3)	4.913	0.027

*SMA* superior mesenteric artery, *LRV* left renal vein

**Table 7:** Treatment modality (N = 110)

Treatment modalities	N (%)
Conservative treatment	71 (64.5)
Endovascular bare stent after 2 months of follow-up	2 (1.8)
Non-conservative treatment	39 (35.5)
Endovascular bare stent	31 (28.2)
Balloon dilation assisting bare stent	8 (7.3)
Coil assisting bare stent	1 (0.9)
Interventional thrombolysis	4 (3.6)
Open surgical treatment	4 (3.6)
Bare stent assisting surgical treatment	1 (0.9)

## Discussion

The SMA, which is the second of the major anterior branches of the abdominal aorta, supplies blood to organs from the lower part of the duodenum through two-thirds of the transverse colon [9]. The SMA is the most frequent site of isolated dissection among the visceral arteries [12]. SISMAD is considered to be an uncommon vascular disease, with abdominal pain as the main symptom. Although there have been some reports on SISMAD, they have focused mainly on its treatment, and the number of cases has been limited (fewer than 45) [7, 14, 16, 17, 20–22]. The number of case in the present study is relatively large, at 110. In addition, SISMAD, as a very rare disease, should be considered in the differential diagnosis when patients have unexplained abdominal pain, which is one of the most common symptoms in the emergency department. However, due to its complexity and rarity, its clinical characteristics, laboratory data, imaging findings, and diagnosis or misdiagnosis have not been investigated in detail. Moreover, in this study, we not only emphasized the aforementioned factors but also found that the maximum SMA diameter and its location on plain CT were essential in diagnosing SISMAD and choosing an appropriate imaging modality.

SISMAD is more prevalent in males in the 5th decade of life. The pathogenesis of SISMAD has been undetermined in most reported cases and has yet to be fully elucidated [4, 9, 23, 24]. Some studies have noted associations with hypertension, smoking, alcohol abuse, trauma, atherosclerosis, cystic medial necrosis, connective tissue diseases and fibromuscular dysplasia [6, 17, 25]. Fibromuscular dysplasia was also associated with renal artery dissection and aortic dissection [26, 27]. In our study, 39.1%, 30.9%, and 41.8% of patients had a history of hypertension, alcohol consumption, and smoking, respectively. Notably, only 1.8% of patients had a history of hyperlipidaemia; however, approximately 1/3 of patients showed hyperlipidaemia on laboratory tests for the first time, which was in line with the result reported by Xu et al. [17], who demonstrated that 45.2% of patients had hyperlipidaemia. It is generally accepted that hypertension was the most significant comorbidity [4, 6, 13, 17, 28]. Unlike in other vascular atherosclerotic diseases, the prevalence of diabetes mellitus in SISMAD is relatively low [28]. There were no cases of diabetes mellitus in the present study, which was in accordance with studies by Mkangala et al. [2], Zhang et al. [12], and Han et al. [29]. In addition, celiac artery stenosis or occlusion caused by celiac trunk dissection and compensatory increased flow of the SMA can lead to weakening of the arterial wall by increasing the haemodynamic shearing forces. This may be another possible mechanism of the disease [25]. In our study, 7.3% of cases were complicated with celiac trunk dissection. Importantly, mechanical stress on the arterial wall at the inferior pancreatic edge is an important aetiology [11, 23, 24]. Mechanical stress is also associated with the aortomesenteric angle, which is larger in patients with than without SISMAD [30]. Kim [24] mentioned that mechanical stress was caused by the transition of the SMA at the lower margin of the pancreas from a fixed to a relatively mobile state. Park et al. [31] also suggested mechanical stress induced by convex curvature as an aetiology.

Autopsy studies have shown that the incidence of SMA dissection is 0.09% [32], and we found that the number of cases increased over time at our hospital (Fig. 1). The incidence of SISMAD is likely underestimated because of the lack of reliable clinical signs and laboratory findings. Clinically, manifestations can vary and may be nonspecific [8]. The presentation varies from incidental discovery on CTA without symptoms to acute pain [12]. In symptomatic patients, the common clinical manifestations are acute pain, such as abdominal pain, back pain and chest pain, with abdominal pain being the most common, occurring in 72.2–100% of cases [4, 5, 7, 22, 28, 29]. Park et al. [31] reported that 65.8% of patients presented with epigastric/periumbilical symptoms, while 15.8% had postprandial aggravation. Kwon et al. [16] reported that the mean initial abdominal visual analogue pain score was 7 (range, 5–9). Park et al. [31] mentioned that the pain score was 7–10 in 78.9% of patients. The pain may be related to stenosis of the true lumen, the dissection length, rupture of the dissection, inflammation around the SMA stimulating the visceral nerve plexus, peritonitis or bowel ischaemia [11, 12, 24, 25]. Apart from the above, aberrant haemodynamic forces due to the convex morphology of the SMA, particularly 1.5–3 cm from the origin, may play a role in abdominal pain [7].

SISMAD is usually seen in middle-aged males presenting with acute or chronic epigastric or upper left quadrant pain. SISMAD should be suspected in all patients presenting with intractable abdominal pain or other common causes of unexplained acute abdomen and one or more risk factors for vascular atherosclerotic disease [4]. CECT or CTA is the diagnostic test of SISMAD. Luan et al. [5] investigated 589 Chinese SISMAD patients and found that 95.2% were diagnosed by CECT. Ullah et al. [4] performed a meta-analysis involving 145 cases and showed that 35.8% were diagnosed by CECT. The authors emphasized that the higher incidence of SISMAD was likely due to the introduction of CECT for investigating abdominal pain, which resulted in an earlier diagnosis [4]. Typical CECT findings include the characteristic double-lumen sign of the SMA. Further examination by CTA is suitable and provides a three-dimensional view of the luminal borders and extraluminal organs. Given the presentation of SISMAD as acute or chronic abdominal pain, a common but nonspecific complaint encountered in the emergency room, it remains essential that all medical professionals be aware of SMA dissection as a possible underlying aetiology. In short, when unexplained abdominal pain is encountered in the emergency room, CECT or CTA should be performed without a doubt to diagnose or rule out SISMAD.

As a rare disease, SISMAD is often misdiagnosed or missed. Ullah et al. [4] reported that some patients who presented with acute abdominal pain and underwent imaging tests were misdiagnosed with gastroenteritis, gastric pain, or nonspecific pain. Ullah et al. [4] reported that 7 cases (4.8%) were discovered either on autopsy or incidentally on CT performed for pancreatitis or other reasons. Zhao et al. [10] investigated 11 SISMAD patients who were admitted to the emergency department and found misdiagnosis or missed diagnosis in 7 (63.6%) of these cases; 2 patients were misdiagnosed with gastroenteritis, 1 with appendicitis, 1 with myocardial infarction, and 1 with intestinal obstruction, while 2 were considered to have unknown maladies. In our study, there were 32 cases (29.1%) of misdiagnosis or missed diagnosis. SISMAD patients, especially those with Yun type IIb SISMAD, could be misdiagnosed with SMA embolism since these conditions have the same manifestations of CT and are both acute-onset diseases that usually require emergency surgery [12]. In our study, Yun type IIb was the most common type of SISMAD, accounting for 63.6%, which was in accordance with the studies by Mkangala et al. [7], Kwon et al. [16], and Li et al. [22]. Twenty cases of SISMAD were misdiagnosed as SMA embolism. Among them, there were 15 cases of Yun type IIb SISMAD, including 8 cases in which the longitudinal sections on CECT or CTA were similar to that shown in Fig. 3G. Hence, we should bear in mind that Fig. 3G shows SISMAD, not SMA embolism, which are easily confused by emergency doctors and radiologists. It is clear that SISMAD is easily misdiagnosed and missed. There are several reasons for this outcome, as follows: (i) the disease is very rare, and both doctors and radiologists lack awareness; (ii) there are no reliable clinical signs or laboratory findings [5] (approximately 1/2 of patients had a slightly increased C-reactive protein level and white blood cell count, and 1/3 had an increased D-dimer level in our study); and (iii) some patients have other diseases of acute abdomen simultaneously, such as urinary calculi, intestinal obstruction, and gallstone/cholecystitis.

To improve the diagnostic rate, reduce the misdiagnosis rate, and optimize testing, it is important to measure the maximum diameter of the SMA and locate its site in patients with persistent or aggravated abdominal pain. The findings of plain CT often are negative or are positive but cannot explain the symptoms. The normal diameter of the SMA is 6–8 mm [13]. Kim et al. [14] measured the maximum SMA diameter in 22 SISMAD patients with partial or complete thrombosed false lumen and showed that the mean value was 11.6 mm. Yan et al. [15] used plain CT to measure the diameter of the SMA and found mean values in the SISMAD group (n = 20) and the control group (n = 20) of 11.69 ± 1.26 and 7.10 ± 0.97 mm, respectively. We used plain CT to measure the SMA diameter in 66 SISMAD patients and found that the mean maximum diameter of the SMA was 12.1 mm. We used CECT or CTA to measure the SMA in all SISMAD patients, resulting in a mean value of 13.0 mm. Importantly, the location of the maximum SMA diameter in approximately 2/3 of patients was on, not above, the LRV plane. Generally, from the ostium to the distal SMA, the lumen becomes smaller. Hence, in patients with abdominal pain that remains unexplained after plain CT, if the maximum SMA diameter is more than 12 mm or the location of the maximum diameter is on or below the LRV plane, we highly recommended mesenteric CTA as the first examination to diagnose SISMAD. Otherwise, CECT should be considered the first examination to rule out SISMAD.

The treatment regimen for SISMAD is still not well established, and there are different approaches, including conservative treatment, endovascular treatment, interventional thrombolysis and open surgical treatment. Conservative treatment was used as the first-line therapy for symptomatic patients, as recommended by the European Society of Vascular Surgery guidelines [21, 31, 33]. Conservative treatment includes blood pressure control, bowel rest with fasting, anticoagulation, and antiplatelet treatment. Karaolanis et al. [28] performed a meta-analysis and found that 438 cases (72%) of symptomatic SISMAD were managed conservatively. Conversion from conservative treatment to endovascular and open surgical treatment was required in 12.3% and 4.4% of patients, respectively. In our study, 71 cases (64.5%) were managed conservatively, and 2 patients underwent endovascular bare stent implantation after 2 months of follow-up. In our study, there were differences between the conservative and non-conservative groups in the residual true lumen diameter or degree of true lumen stenosis and the presence of intestinal obstruction or bowel necrosis. Severe stenosis of the true lumen is also associated with bowl ischaemia [11]. The residual true lumen diameter was significantly better in the conservative group than in the non-conservative group, which was in accordance with a study by Li et al. [20]. It is generally accepted that endovascular or surgical therapy should be considered if conservative treatment fails or the condition is complicated with signs of bowel infarction [1, 9, 21]. Compared with conservative treatment, endovascular treatment has been associated with a higher rate of SMA remodelling and a lower rate of cumulative event-free survival in the long term [1, 34].

### Limitations

Our study is limited by the fact that it was a an 8-year retrospective study. Therefor, some patients may differ in imaging quality and section thickness, which may induce measuring bias; some patients did not have complete laboratory data or imaging data. Besides, even though we emphasized the importance of the maximum SMA diameter and its location on plain CT in selecting the imaging modality for diagnosing SISMAD, the study lacked a comparison of the maximum SMA diameter and its location on plain CT between SISMAD patients and non-SISMAD patients who were admitted to the hospital complaining of abdominal pain in the same period.

## Conclusion

SISMAD is a rare disease presenting with abdominal pain, which is more prevalent in men in the 5th decade of life. Many patients had a history of hypertension, but not diabetes. Laboratory data lack of specificity and most of the location of the maximum SMA diameter was on the LRV plane. Insufficient awareness and disease features were the main reason for misdiagnosis and missed diagnosis. Hence, it is vital to create awareness that SISMAD should be considered in the differential diagnosis of patients presenting with unexplained abdominal pain, especially males, those in their 5th decade of life, those with hypertension, and those with an enlarged SMA diameter or a maximum SMA diameter located on the LRV plane. Mesenteric CTA or CECT should be recommended for the investigation of these conditions. Yun type IIb has several subtypes. It is the most common type and easily misdiagnosed.

## Data Availability

The datasets used and/or analysed during the current study available from the corresponding author on reasonable request.

## Abbreviations

SMA: Superior mesenteric artery
SISMAD: Spontaneous isolated superior mesenteric artery dissection
CT: Computed tomography
LRV: Left renal vein
CECT: Contrast-enhanced computed tomography
CTA: Computed tomography angiography
DSA: Digital subtraction angiography
